# Skin cancer risk in hereditary mixed cancer syndromes

**DOI:** 10.1186/s13053-025-00326-7

**Published:** 2025-12-02

**Authors:** Veera Nikkola, Anna Alakoski, Jukka-Pekka Mecklin, Toni T. Seppälä, Jussi Nikkola, Kashmintan Schrader

**Affiliations:** 1 Hereditary Cancer Program, BC Cancer, 600 West 10th Avenue, Vancouver, Canada; 2 Department of Medical Genetics, University of British Columbia, Vancouver, Canada; 3 Department of Dermatology, Tampere University Hospital, Tampere, Finland; 4 Department of Education and Research, The Wellbeing Services County of Central Finland, Jyväskylä, Finland; 5 Faculty of Medicine and Health Technology, Tampere University and Tays Cancer Centre, Tampere, Finland; 6 Vancouver Prostate Centre, Department of Urologic Sciences, University of British Columbia, Vancouver, Canada; 7 Sport and Health Sciences, University of Jyväskylä, Jyväskylä, Finland

**Keywords:** Hereditary cancer syndromes, Hereditary skin cancer, Germline cancer susceptibility mutations, Genetic cancer predisposition, Skin cancer screening

## Abstract

Hereditary cancer syndromes are genetic conditions that increase an individual’s risk for multiple cancer types, often due to mutations that affect critical cellular processes such as DNA repair and cell cycle regulation. Skin cancers, including malignant melanoma (MM), basal cell carcinoma (BCC), squamous cell carcinoma (SCC), and related precancerous lesions may be underrecognized in some hereditary cancer syndromes, as suggested by underlying biological mechanisms and their underreporting in studies. In this narrative review, we examine the skin cancer risks associated with the most prevalent hereditary cancer syndromes, including Li-Fraumeni syndrome (LFS), Lynch syndrome (LS), hereditary breast and ovarian cancer syndrome (HBOC), ATM-associated hereditary cancer syndrome, CHEK2-associated hereditary cancer syndrome, BRIP1-associated cancer predisposition, and hereditary leiomyomatosis and renal cell carcinoma (HLRCC). This review consolidates existing evidence and suggests that mixed cancer syndromes, especially LFS, LS, and HBOC but also pathogenic *ATM* and *CHEK2* variants may predispose individuals to skin cancers, warranting tailored screening and preventive measures. On the basis of emerging evidence, we recommend dermatologic evaluation and individualized UV protection strategies for patients with reviewed hereditary cancer syndromes to reduce skin cancer risk and enhance early detection.

## Introduction

The presence of multiple cancers in an individual or family, especially at a young age, suggests an underlying genetic cause [1]. In this review, we use the term *hereditary mixed cancer syndrome* to describe conditions that extend beyond a single tumor type and involve primarily non-cutaneous cancers, while acknowledging their genetic and biological heterogeneity. This terminology is intended to distinguish these syndromes from primarily cutaneous cancer syndromes, such as CDKN2A-related melanoma predisposition, oculocutaneous albinism, and basal cell nevus syndrome, which have been extensively reviewed elsewhere [2, 3]. Hereditary mixed cancer syndromes involve genetic predispositions to multiple cancer types, often due to mutations in genes involved in DNA repair, cell cycle regulation, or apoptosis. Genetic variants associated with these syndromes are classified by the American College of Medical Genetics and Genomics (ACMG) into categories on the basis of pathogenicity: pathogenic, likely pathogenic, variant of uncertain significance, likely benign, and benign [4]. At least 2% of unaffected individuals carry germline pathogenic variants linked to hereditary cancer syndromes, with higher rates in founder populations [5].

**Fig. 1:**
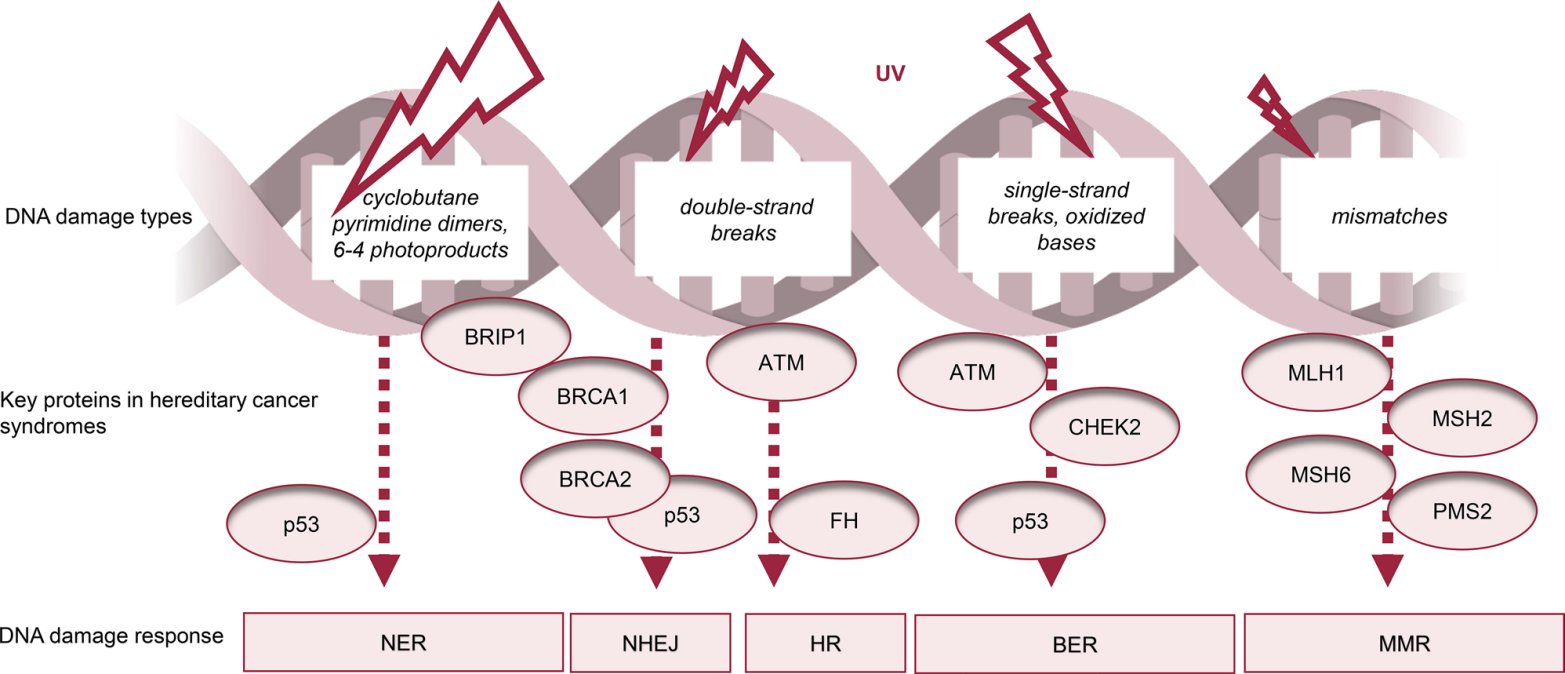
Ultraviolet radiation (uvr) induced dna damage mechanisms and repair pathways in common mixed cancer syndromes. UVR-induced DNA lesions are primarily cyclobutane pyrimidine dimers and 6–4 photoproducts. Additionally, UVR can generate reactive oxygen species, leading to oxidized bases, single-strand breaks, and, in some cases, double-strand breaks due to replication stress. These forms of damage can contribute to replication errors if not properly repaired. The DNA damage response is the process by which cells detect and fix DNA damage to maintain genome stability. The nucleotide excision repair (NER) pathway eliminates cyclobutane pyrimidine dimers, and base excision repair (BER) repairs oxidized bases and single-strand breaks. DNA double-strand breaks are the main trigger for activating ATM protein kinase. This activation triggers a cascade of events that regulate DNA repair, cell cycle progression, and apoptosis. In the context of oxidative stress, ATM plays a supportive and regulatory role by activating various cellular pathways, including p53-dependent regulation of BER components. Mismatch repair (MMR) proteins correct replication errors. Non-homologous end joining (NHEJ) is an error-prone repair process that quickly fixes DNA double-strand breaks without a template. In contrast, homologous recombination (HR) is a precise repair mechanism that uses a template for accurate DNA repair, mainly in dividing cells. BRCA1/2 mutations impair HR, leading to increased reliance on error-prone NHEJ. TP53 mutations disrupt HR, NHEJ, and NER, compromising genome stability. FH mutations cause metabolic changes that induce replication stress and reduce HR efficiency. When the BER pathway is disrupted, improperly repaired single-strand breaks accumulate and can degenerate into double-strand breaks, increasing the degree of dependence on alternative repair mechanisms such as HR and NHEJ [7, 8]

Cutaneous malignancies are classified into non-melanoma skin cancers (NMSCs) and malignant melanoma (MM) [6]. Ultraviolet radiation (UVR) induces DNA damage through cyclobutane pyrimidine dimers, 6–4 photoproducts, oxidative stress, and occasional single- and double-strand breaks, potentially leading to replication errors (Fig. 1). In particular, cumulative exposure is a key factor in the development of the most common NMSCs: basal cell carcinoma (BCC) and squamous cell carcinoma (SCC) [9]. While BCC rarely metastasizes, untreated cases can invade deep tissues [10]. High-risk SCC may spread to distant organs [11]. Keratoacanthoma (KA), a well-differentiated SCC, grows rapidly and has distinct histological features [12]. Premalignant lesions such as actinic keratoses (AKs) and morbus Bowen (MB) can progress to SCC [13]. Rare NMSCs include sebaceous gland carcinoma (SGC) and cutaneous leiomyosarcoma (cLMS), among others [14]. Cutaneous MM originates from melanocytes, and genetic factors and childhood sunburn contribute to the risk of MM [15]. Early detection is crucial, as advanced cases can be fatal [15]. Melanoma in situ (MIS) is a precursor to MM [16].

NMSCs are the most common malignancies among Caucasians and are steadily increasing worldwide, placing a major burden on healthcare systems [17]. In 2022, Ireland had Europe’s highest NMSC incidence at 37.7 per 100,000 [18], whereas North America reported rates of 53.3 per 100,000 in Canada and 63.3 per 100,000 in the United States [19]. MM incidence is also increasing, peaking in Australia/New Zealand at 41.6 per 100,000 [20], with Scandinavian countries quickly catching up [19]. This increasing prevalence reflects both genetic susceptibility to UVR and demographic shifts, as populations in these regions, which are largely composed of fair-skinned individuals, continue to age [21]. In dark-skinned individuals, UV-blocking eumelanin scavenges free radicals, whereas the lighter pheomelanin in light-skinned individuals generates reactive oxygen species upon UV exposure [22, 23]. By absorbing UVR, pheomelanin produces free radicals that cause cellular damage, making lighter phototypes more prone to skin cancer [24].

As skin cancer is a growing problem worldwide and few guidelines exist for its risk management in hereditary mixed cancer syndromes [25, 26], this review evaluates common skin cancers, precancerous lesions, and selected rare NMSCs within these syndromes (Table 1). Based on their prevalence in previous studies [27–36] and our clinical experience in dermatology, we focus on Li-Fraumeni syndrome (LFS), Lynch syndrome (LS), hereditary breast and ovarian cancer (HBOC) syndrome, ATM-associated hereditary cancer syndrome, CHEK2-associated hereditary cancer syndrome, cancer predisposition associated with *BRIP1* mutations, and hereditary leiomyomatosis and renal cell carcinoma (HLRCC) in this narrative review. This review also provides surveillance suggestions for hereditary cancer syndromes.

**Table 1:** The most common hereditary cancer syndromes potentially associated with skin cancers

Hereditary cancer syndrome	Germline pathogenic variant	Prevalence	Penetrance (other than skin cancers)	Altered DNA repair mechanism (Fig. 1)	Most significant associated other than skin cancers
Li-Fraumeni syndrome (LFS)	*TP53* heterozygous	1 in 3,500 [27]	high	HR-DDR and defects in NER, NHEJ	Sarcoma, brain, breast and adrenocortical
Lynch syndrome (LS)	MLH1, MSH2, MSH6, PMS2, EPCAM** heterozygous	1 in 300 [28]	high	MMR	Colorectal, endometrial, urothelial, ovarian, gastric, small intestine
Hereditary Breast and Ovarian Cancer syndrome (HBOC)	*BRCA2* heterozygous	1 in 400 [29] *	high	HR-DDR	Breast, ovarian, pancreatic, prostate
	*BRCA1* heterozygous	1 in 500 [30, 31] *	high	HR-DDR	Breast, ovarian, pancreatic
ATM-associated hereditary cancer syndrome	*ATM* heterozygous	1 in 100 [32]	moderate	HR-DDR	Breast, pancreatic, prostate
	all truncating variants	1 in 500 [33]	moderate	HR-DDR	Breast, pancreatic, prostate
CHEK2-associated hereditary cancer syndrome	*CHEK2* heterozygous	1 in 164 [34]	moderate	HR-DDR	Breast, prostate, kidney
	all truncating variants	1 in 189 [35]	moderate	HR-DDR	Breast, prostate, kidney
	c.1100delC	1 in 214 [33]	moderate	HR-DDR	Breast, prostate, kidney
BRIP1-associated cancer predisposition	*BRIP1* heterozygous	1 in 700 [35]	moderate	HR-DDR	Ovarian, breast
Hereditary Leiomyomatosis and Renal Cell Carcinoma (HLRCC)	*FH* heterozygous	1 in 500 [36]	high	HR-DDR, Krebs cycle	Kidney

HR-DDR: homologous recombination DNA damage repair. A mechanism to repair DNA double-strand breaks through the homologous recombination (HR) repair pathway. MMR: mismatch repair, NER: nucleotide excision repair, NHEJ: non-homologous end joining

^*^The prevalence of *BRCA1*- and *BRCA2*-associated HBOC is greater in certain populations because of founder variants

^**^EPCAM deletions silence MSH2, leading to a Lynch syndrome phenotype

## Li‒Fraumeni syndrome

Li‒Fraumeni syndrome (LFS) is a rare mixed cancer syndrome that is inherited in an autosomal dominant fashion and is caused by a germline mutation in the *TP53* gene [25]. The gene encodes the transcription factor p53, which is involved in regulating cell growth and apoptosis and maintaining genomic stability. The clinical definition of LFS relies on an individual’s personal history and/or family history of cancer. The cumulative lifetime cancer incidence in this syndrome is nearly 100% [37]. Characteristic LFS tumors include sarcoma, brain tumor, breast cancer and adrenocortical carcinoma [38].

MM was first proposed as part of the LFS cancer spectrum in 1987 when it was reported in seven families with a syndrome described by Li and Fraumeni nearly two decades earlier [39, 40]. However, a later study of 738 primary cancers in *TP53* mutation carriers and their first-degree relatives revealed only six cases of MM (0.8%), leading researchers to question its inclusion in LFS [41]. This study had limitations, including unconfirmed *TP53* mutation status in participants and a lack of a proper control group. The ages at cancer diagnosis were instead compared with data from the general population. In confirmed LFS patients, the median age at MM diagnosis was 35 years, whereas in the general population, it was 54 years [41]. Since then, skin cancers have been reported in studies with confirmed germline *TP53* mutations. A retrospective cohort study revealed 1 MM (0.7% of all primary cancers) and 4 other skin cancers (2.8%) among 91 LFS patients [38]. A larger prospective cohort reported 9 NMSC cases and 3 MM cases as first cancers in 286 patients, with 7 NMSC cases and 6 MM cases as second cancers [37]. In a retrospective cohort of 322 mutation carriers who were originally selected on the basis of clinical LFS criteria, 2.4% had MM, and 1.6% had other skin cancers [42].

The risk of skin cancers in LFS patients was first detailed in a study of 71 unselected *TP53* mutation carriers, which revealed a significantly greater cumulative risk of BCC and MM than did the general Dutch population, although SCC risk was not assessed due to the limited number of cases. Premalignant lesions such as dysplastic nevi, AK, and MB were found in 6% of LFS patients who later developed skin cancer [43]. In a cohort of 483 LFS individuals from 145 families, 113 cutaneous malignancies were identified, with cumulative incidences of 14.9% for MM and 23.4% for BCC/SCC by age 70. However, only 64% of the cases were confirmed, and the rest were self-reported, suggesting that some may have been premalignant. The median ages at diagnosis were 40 years for BCC, 53 years for SCC, and 42 years for MM, all of which are younger than those in the general population [44]. While no childhood MM, BCC, or SCC cases have been reported in cohort studies [42–44], at least two childhood MM cases with LFS have been reported [41, 45]. The risk of sarcomas in LFS patients appears to extend to the skin, including an increased risk of cLMS [44]. These findings underscore the need for ongoing surveillance and early detection strategies for skin cancers in individuals with LFS.

## Lynch syndrome

Lynch syndrome (LS), with a prevalence of 1 in 300, is a common autosomal dominant hereditary cancer syndrome [26]. It predisposes individuals to various abdominal cancers due to mutations in DNA mismatch repair genes *(MLH1, MSH2, MSH6, PMS2*) or *EPCAM*, leading to microsatellite instability detectable in tumors [24, 26]. LS patients have a high cancer risk, particularly in organs with high cell turnover, such as the colon and endometrium [46]. The skin’s rapid turnover may also increase cancer risk due to increased DNA replication errors, which LS patients repair less effectively. However, unlike all other cancers, skin cancer risks have not been systematically reported in the Prospective Lynch Syndrome Database [47]. Biallelic pathogenic variants in LS genes cause constitutional mismatch repair deficiency (CMMRD), a rare syndrome with limited life expectancy, in which SCC at age 17, MM at age 16, and two sebaceous tumors have been reported [48–50].

Muir-Torre syndrome (MTS), a subtype of LS, is characterized by sebaceous gland tumors (e.g., SGC) and an associated visceral malignancy [51–53]. It is considered a distinct entity within LS due to unique skin tumors linked to MMR deficiency rather than simply a variation in which some LS patients develop skin cancers [53, 54]. In MTS, skin manifestations are the most common type of tumor and can appear up to 25 years before visceral malignancy [54].

MMR immunohistochemistry (IHC) can screen for LS in skin neoplasms, although its sensitivity and specificity are not ideal [24, 55]. Genetic testing should be prioritized, particularly in cases where SGC(s) occurs at a young age or below the head and neck area, even in the absence of visceral malignancy [56]. KAs are common in MTS, especially those with sebaceous differentiation [54, 57]. In MTS, KA or other well-differentiated SCCs sometimes exhibit atypical features suggestive of progression toward more aggressive SCC [58].

The *MSH2* gene is the most commonly mutated LS gene in MTS, and individuals with LS carrying *MLH1* or *MSH2* mutations are significantly more likely to develop skin cancer than those with *MSH6* or *PMS2* mutations [59, 60]. *EPCAM* mutations silence *MSH2*, with likely similar cancer risks [24]. A review of 607 LS patients revealed that 9.2% had sebaceous neoplasms or KAs, which were strongly correlated with *MLH1* and *MSH2*/*EPCAM* variants, with skin cancers reported as early as age 25 [61]. CMMRD due to *MLH1* or *MSH2* mutations is extremely rare, with a life expectancy of only 6 to 9 years and no reported skin cancers [48].

Links between SCC, MB, AK, and MTS/LS have been documented in case reports, small series, and one chart review. Case reports have described SCC in a sun-protected area of a 55-year-old LS patient with an *MLH1* mutation [62] and on the nose of a 41-year-old MTS patient with an *MSH2* deletion [63]. In an *MSH2*-mutated family, the mother (LS) had AK and MB, whereas the daughter (MTS) developed KA [64]. Among the 28 skin lesions from 17 MTS patients, 3 KAs and 1 SCC were reported, whereas the other tumors were sebaceous [65]. Among 331 LS patients, the cumulative SCC/sebaceous carcinoma risk reached 7.8% by age 70, whereas it reached 1.29% in the general Dutch population [66]. Importantly, established skin cancer risk factors did not correlate with malignant skin tumor development [66].

MMs from nine patients in eight LS families were analyzed in a registry-based study, including one MIS, one nodular MM, and several superficial spreading MMs. The tumor locations resembled sporadic MM, but the median diagnosis at 46 years was notably younger [67]. However, a cohort study of 331 LS patients from 194 families revealed no increased risk of MM or BCC [66]. More recently, a prospective study of 400 MM patients with a personal or family history of cancer identified germline *MLH1* and *MSH2* pathogenic variants in several cases, none of which were present in the gnomAD control group [68].

The only systematic review on LS-related skin cancers analyzed 380 confirmed cases, although it was largely based on single case reports with incomplete lesion documentation. As expected, based on earlier studies, SGC, SCC, and BCC were the most common cancers, with MSH2 variants being the most frequent genotype [69]. Interestingly, unlike in the general population, SCC appeared more common than BCC. [69]. The risk of squamous malignancies in LS patients is likely underrecognized [70]. While LS is strongly linked to SGC and KA, its association with advanced SCC remains uncertain. The evidence for MM in LS is contradictory.

## Hereditary breast and ovarian cancer syndrome

*BRCA1/2* mutations, which are inherited in an autosomal dominant manner with a prevalence of 1 in 300–500, increase the risk of breast, ovarian, peritoneal, prostate, and pancreatic cancers [1, 23, 27]. Despite high penetrance, risk varies even within families. These mutations disrupt homologous recombination DNA damage repair (HR-DDR), forcing cells to rely on error-prone pathways like non-homologous end joining (NHEJ), ultimately leading to genomic instability [71, 72] (Fig. 1). BRCA1 also plays a role in UV-induced skin damage, separate from HR-DDR [73]. Biallelic *BRCA1/2* mutations can cause Fanconi anemia, which may increase the risk of early-onset NMSC [74]. Additionally, immunosuppression and bone marrow transplants, which are often part of the management of Fanconi anemia, can further increase the risk [74–76].

The link between MM and germline *BRCA1/2* mutations was initially studied before genetic testing for all family members became routine. Early research, including findings from the Breast Cancer Linkage Consortium, reported a 2.6-fold increased MM risk in suspected *BRCA2* carriers of European and North American descent [77], with similar results in a smaller Swedish study [78] and a large U.K. cohort [79], although the latter two lacked statistical significance. Later studies with confirmed mutations revealed that *BRCA2* c.2971A > G nearly doubled MM risk in the Polish population [80], whereas *BRCA2* c.9976A > T was significantly associated with MM risk in the Swedish population [81]. A prospective study of 6,207 *BRCA1/2* carriers revealed an increased MM risk, with an eight-year cumulative risk of 2.5% for *BRCA1* carriers and 2.3% for *BRCA2* carriers, compared to 1.5% in the general U.S. population [82]. However, a large analysis of 7,618 families in the Consortium of Investigators of Modifiers of BRCA1/2 (CIMBA) revealed no MM associations, possibly due to underreporting in self-reported cancer histories [83].

BRCA1‒p53 interactions play a key role in repairing UV-induced DNA damage, which might lead to increased SCC susceptibility in *BRCA1*-deficient individuals [84, 85]. Observational studies suggest a potential link between *BRCA1/2* mutations and both SCC and BCC. One study of 1,145 individuals from *BRCA1*-associated families reported a 4.8-fold increase in SCC, although the lack of confirmed carrier status may have led to an overestimated risk [78]. Another study reported *BRCA1/2* mutations in 25% of high-risk breast cancer patients with NMSC, with *BRCA1* being more prevalent, but the small sample size of only 16 cases limits its significance [86].

BRCA research often analyzes male and female carriers separately, which can introduce bias. In males, SCC risk appeared to be greater in *BRCA1* families, but the small number of cases resulted in wide confidence intervals [78]. Another study revealed an increased risk of NMSC, particularly BCC, among 950 female *BRCA2* mutation carriers [87]. Recently, a large prospective study of female *BRCA1/2* carriers revealed no increased NMSC risk, although self-reported data may have led to underreporting [82]. Overall, *BRCA* mutations likely predispose individuals to MM, but research on their connection to NMSC is biased and inconsistent due to methodological limitations, including the lack of genetic confirmation and the exclusion of NMSC cases in many studies [77, 79, 83].

## ATM-associated hereditary cancer syndrome

*ATM* occupies a distinct position among hereditary cancer susceptibility genes because it represents a medium-penetrance condition, in contrast to high-penetrance syndromes such as LS, HBOC and LFS. Approximately 1–2% of Caucasian adults in the U.S. carry a pathogenic *ATM* heterozygous variant [30], which is associated with increased risks of breast, pancreatic, prostate, and possibly other cancers [88]. The large size of the *ATM* gene and the prevalence of variants of uncertain significance have limited the identification of additional cancer associations. *ATM* variants follow autosomal dominant inheritance and encode a kinase activated by DNA damage, including UVR—a key factor in skin cancer development [89]. ATM facilitates DNA repair through the HR-DDR pathway, which involves BRCA1/2, BRIP1, p53, and Chk2 [90] (Fig. 1). Biallelic variants cause ataxia telangiectasia (AT). Despite AT skin cells in culture having an altered DNA damage response to UV radiation [91], AT does not appear to increase skin cancer risk, possibly because of UVR-induced enhanced skin pigmentation, limited life expectancy, and physical limitations [92, 93].

In a cohort study, *ATM* pathogenic variants were 2–3 times more common in MM patients (*n* = 7,970) than in gnomAD non-Finnish European controls [94]. Additionally, in a separate cohort of 2,104 MM patients from Europe, the U.S., and Australia, loss-of-function variants in *ATM* were nearly three times more common than in gnomAD. The allele frequency in the MM cohort was 0.5%, whereas it was 0.2% in gnomAD [95]. The *ATM* allele c.5750 G > C, which is linked to hereditary breast and ovarian cancers, was also more common in the MM cohort. NMSCs were found to be common, with BCC being the most common cancer after MM, but their frequency was not compared with that of healthy controls [95]. Population stratification bias may have influenced the results.

In a case‒control study consisting of 627,742 patients referred for hereditary cancer testing, the risk of cutaneous MM was also slightly elevated (OR 1.46) in carriers of pathogenic *ATM* variants [88]. This finding highlights that even though *ATM* pathogenic variants are likely predisposed to MM, risk might have been overestimated in studies comparing cohorts with a sample of the general population.

A recent case report described a Hispanic family with the *ATM* c.3747-1 G > C variant, where members with skin phototype IV developed MM, including one diagnosed at age 24 [96]. Valuable data to include MM in the spectrum of cancers associated with *ATM* mutations were also obtained when tissue samples from fourteen MM patients with germline heterozygous *ATM* variants classified as pathogenic or variants of uncertain significance were investigated. In two of the three patients with pathogenic variants, the ATM protein was absent from the MM tissue, which supports the hypothesis that the loss of ATM’s tumor suppressor function may contribute to MM development [97]. Despite the relatively small sample size, the study clearly demonstrated a two-hit scenario, a typical mechanism for hereditary cancer syndromes, occurring within *ATM* mutations in MM.

Research has indicated that *ATM* heterozygotes might carry a moderate causal risk for MM. Future studies should focus on larger cohorts and more comprehensive tumor analyses to further clarify the role of *ATM* mutations in MM development. As more individuals with AT are identified, systematically collecting data on skin cancer in both classic and variant AT is essential.

## CHEK2-associated hereditary cancer syndrome

Checkpoint kinase 2 (CHEK2) is crucial for maintaining the integrity of the DNA damage response during the cell cycle. It is activated by signals from ATM and ATR proteins during double-strand DNA breaks caused by UV radiation or other stressors, triggering downstream effectors such as p53 and BRCA1 [98] (Fig. 1). Germline *CHEK2* mutations, which are inherited in an autosomal dominant manner, have a variable prevalence: from 1 in 100 to 1 in 1000 in different populations [99]. The most frequent truncating variant, c.1100delC, has prevalence rates ranging from 0.5 to 1.5% in the general population [31, 100, 101]. *CHEK2* mutations are associated with breast, prostate, kidney, and possibly other cancers, but the presence of variants of uncertain significance and variable penetrance complicates the confirmation of their role in specific cancers [99]. Rare biallelic *CHEK2* mutations appear to increase the overall cancer risk, although a statistically significant increase in skin cancer risk has not been reported [102, 103].

Studies on the link between CHEK2 and skin cancers have yielded mixed results. The c.1100delC variant has been associated with impaired repair of UV-induced DNA double-strand breaks [104]. A robust meta-analysis involving 2,619 MM patients and 17,481 controls revealed that carriers of this variant had a 1.8-fold increased risk of MM [105]. However, three smaller cohort studies, including 630 unselected MM patients [80], 101 MM patients [106], and 46 patients with three or more primary MMs [107], did not find a significant association between pathologic *CHEK2* variants and MM. A rare *CHEK2* variant c.349A > G has been reported in a patient with multiple primary MMs, although this could also represent a coincidental finding [108]. The only study on *CHEK2* and NMSC focused on BCC, finding a significant enrichment of *CHEK2* mutations in patients with six or more BCCs compared with the ExAC non-Finnish European population, although only two mutations were identified owing to the small sample size [109]. No studies have fully explored the link between *CHEK2* and SCC, although one study reported a biallelic homozygous *CHEK2* c.1100delC carrier who developed SCC at age 67 and MB at age 66 [102].

The role of CHEK2 in skin cancer remains uncertain. One large study and molecular mechanism support a greater risk of MM in c.1100delC carriers, whereas other studies remain inconclusive. Limited research on BCC has revealed a possible connection, and no studies have explored SCC.

## BRIP1-associated cancer predisposition

*BRIP1* encodes FANCJ (also known as BRIP1), a protein crucial for the double-strand break repair function of BRCA1 and the DNA damage response (Fig. 1). Germline *BRIP1* mutations increase the risk of tubo-ovarian and possibly breast cancer [33, 110]. The prevalence of heterozygous *BRIP1* mutations is approximately 1 in 700 in the general population [33]. Biallelic *BRIP1* mutations can cause Fanconi anemia, possibly increasing early-onset NMSC risk, as in the case of *BRCA* mutations [74–76].

The *BRIP1* germline mutation was first linked to skin cancer in a family with a rare, likely pathogenic variant associated with MM. Despite no evidence of loss of heterozygosity in tumor samples, the *BRIP1* c.2543 G > A mutation was found in five family members, three of whom were diagnosed with MM and one with two SCCs. Other cancers, such as liver and ovarian cancers, were also present in the family [81]. Similarly, an 11-year-old patient with spitzoid MM was found to have a heterozygous germline *BRIP1* pathogenic variant c.2053C > T inherited from a mother with no history of MM. This MM harbored both a somatic *BRIP1* variant and an ALK rearrangement [50]. In the Gross Family Melanoma Registry cohort, the incidence of pathogenic germline *BRIP1* variants was significantly greater in MM patients than in gnomAD, further supporting *BRIP1* as a predisposing mutation [68].

Recent studies suggest that *BRIP1* mutations may predispose to MM, although research on this topic is limited. However, no studies have examined the role of BRIP1 in NMSC. Given the potential early-onset NMSC risk associated with biallelic variants, further research is essential.

## Hereditary leiomyomatosis and renal cell cancer

Hereditary leiomyomatosis and renal cell cancer (HLRCC) is an autosomal dominant condition caused by heterozygous mutations in the fumarate hydratase (*FH*) gene. Two large cohorts presented germline *FH* mutation frequencies of 1 in 835 (*n* = 60,706) and 1 in 393 (*n* = 2,504), which were higher than previously reported [34]. Individuals with HLRCC are at significant risk for renal cell carcinoma and commonly develop cutaneous and uterine leiomyomas. The prevalence of cutaneous leiomyomas ranges from 46% to 76%, with higher rates in older individuals. These lesions typically appear at approximately 25 to 30 years of age and increase in size and number with age [111–115]. Biallelic *FH* mutations cause fumarase deficiency (FD), a rare and severe metabolic disorder without a cancer predisposition [116].

MM is not commonly associated with HLRCC, although a retrospective cohort of 56 patients revealed two cases of invasive MM [117]. The sisters with the *FH* c.952C > T mutation were both treated for MIS and BCC of the head area [118]. One MIS was diagnosed at age 44, and the other was diagnosed at 74, with common MM locations in the leg and shoulder [118–120]. Cutaneous leiomyomas carry a risk of transformation into cLMS, although the exact magnitude remains unknown. In a cohort of 182 individuals from 114 HLRCC-affected families, three cLMSs were documented [111]. Similarly, a review of 672 HLRCC patients, including cohort studies and case reports, identified five cLMSs [121]. CLMS are the primary malignant skin cancers in HLRCC. While MM is not typically associated with HLRCC, a few cases of invasive MM and MIS with the presence of BCCs highlight the need for further research on the relationship between HLRCC and skin cancers.

## Discussion

**Fig. 2:**
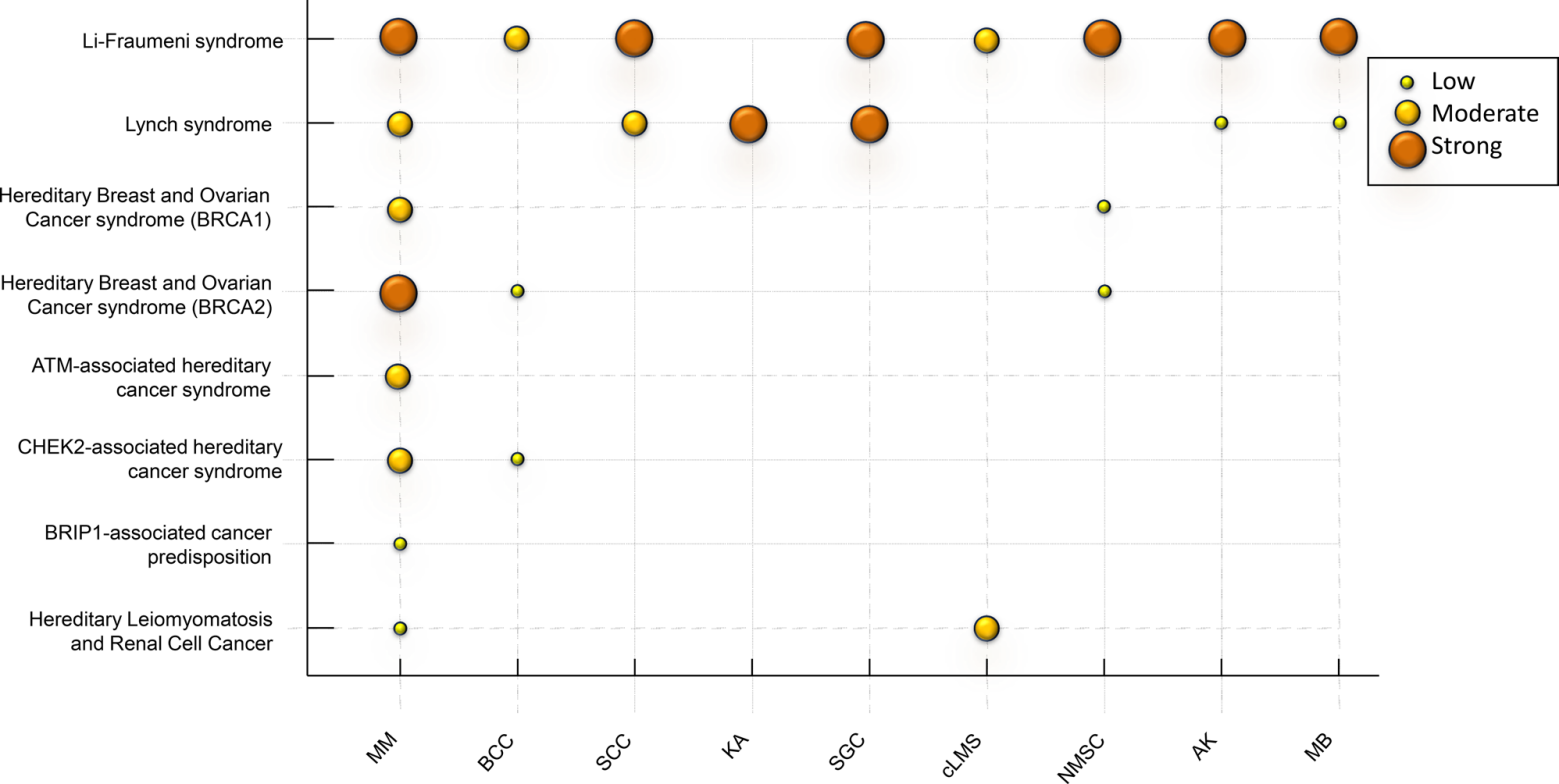
Level of evidence connecting skin cancer risk and mixed cancer syndrome mm: malignant melanoma, SCC: squamous cell carcinoma, BCC: basal cell carcinoma, ka: keratoacanthoma, AK: actinic keratoses, MB: morbus Bowen, cLMS: cutaneous leiomyosarcoma, SGC: sebaceous gland carcinoma, NMSC: non-melanoma skin cancers in total

We summarize that the risk of MM is most likely elevated in LFS, LS, and HBOC, with genetic studies also linking MM to other mixed hereditary cancer syndromes. The risk of NMSC is likely elevated in LFS and LS patients and may also be increased in patients with other mixed cancer syndromes, although limited evidence prevents robust conclusions (Fig. 2).

### Melanoma

Germline predisposition in individuals with MM was recently discovered to be more common than previously thought. In the Gross Family Melanoma Registry, 15.3% of participants were diagnosed with a hereditary cancer predisposition [68]. The majority (52%) of identified germline variants were in HR-DDR and LS-associated genes [68] (Fig. 1). While this study exhibits selection bias compared with the general population, it highlights the significance of hereditary mixed cancer syndromes in MM patients from cancer-prone families.

Emerging evidence suggests a link between various hereditary cancer syndromes and MM. Two retrospective cohort studies [42, 43] and one study based on prospective and retrospective data [44] highlight the increased MM risk in LFS patients. Although the evidence linking MM to LS is contradictory, the young age at which LS carriers can develop MM remains an important factor supporting the connection between LS, MM and MIS [67]. Evidence also points to a potential likely link between *BRCA* pathogenic/likely pathogenic variants and MM [77–82]. *ATM* heterozygotes may carry a moderate risk of developing MM [88, 94–97]. Additionally, CHEK2-Associated Hereditary Cancer Syndrome [105, 108] and cancer predisposition associated with *BRIP1* mutations [50, 68, 81] may represent conditions with a potential association with MM risk. MM and MIS have been reported in HLRCC, but the association remains unclear [117–119].

### Non-melanoma skin cancers

SCC accounts for the majority of deaths related to NMSCs [12]. However, research on the connection between SCC and hereditary mixed cancer syndromes remains limited. One exception is LFS, where NMSC and precancerous lesions leading to SCC are overrepresented, although the specific risk of SCC remains unclear [42–44]. Another is the well-established link between KA, SGC and LS, particularly in the context of MTS [51–58]. Advanced SCC, AK, and MB have been linked to LS in case studies, a chart review, and a systematic review of limited reliability [62–64, 66, 69]. However, the observation that SCCs appear more frequently than BCCs in LS patients [69], combined with the absence of typical risk factors for SCC [66], support a germline origin. A significant knowledge gap persists, as research linking SCC with several HR-DDR genes has yet to be conducted.

Current evidence suggests an elevated risk of BCC, the most common NMSC, in patients with LFS [43, 44], and limited evidence supports the connection of *CHEK2* [109] and *BRCA2* [87] with elevated BCC risk. Other mixed cancer syndromes discussed in this review have little to no evidence regarding BCC. However, there may be an association between *BRCA1/2* mutations and NMSCs overall [86, 87], though this could reflect bias from prior radiation treatment and increased surveillance of mutation carriers, and the finding has not been consistent across studies [82]. Larger prospective studies comparing the risk of BCC, SCC, and precancerous lesions in hereditary mixed cancer syndromes with that in the general population are needed.

### Limitations

This review is a narrative synthesis intended to outline current associations and has its limitations. Certain hereditary cancer syndromes may be associated with an increased risk of skin cancer; however, confirming such associations robustly requires prospective and other rigorous study designs and tumor-level analyses, as findings from genetic panels or retrospective data may not provide definitive evidence. Some genetic variants detected in skin cancer patients in panel testing may have been previously considered unrelated to skin cancer, either because they truly are unrelated or because earlier studies lacked sufficient testing to reveal a connection. The apparent overrepresentation of skin cancers in patients with hereditary cancer syndromes may also reflect several biases. In the case of NMSC, closer clinical surveillance can lead to the detection of otherwise unnoticed, slowly growing asymptomatic lesions, while prior radiation therapy may further contribute. For all skin cancers, ascertainment bias may result from preferential recruitment of families with multiple cancers, including sporadic cases, particularly when carrier status is unconfirmed. Moreover, publication bias can further distort the literature.

### Conclusions, screening, and future strategies

Despite possible biases, the two mixed cancer syndromes that most likely predispose individuals to skin cancers are LFS [42–44] and LS [51–58, 62–64, 66–69]. In LFS, as also reflected in the NCCN guidelines [23], annual dermatologic examinations starting at age 18 are suggested, as the risk of MM, cLMS, BCC, SCC, and precancerous lesions is likely elevated. In LS, particularly among individuals with *MLH1*, *MSH2*, or *EPCAM* mutations, close monitoring for MM, BCC, SCC, and precancerous lesions—beyond rare sebaceous manifestations—may be warranted starting at age 18. No childhood skin cancers have been reported in LS patients [69, 122], and they remain rare in LFS patients [41–45]. However, in line with the U.S. Preventive Services Task Force recommendation, we believe that counseling for UVR exposure reduction in children, adolescents, and young adults with either syndrome can promote sun-protective behaviors without appreciable harm [123].

The evidence linking *BRCA1/2*, *ATM*, *CHEK2*, *BRIP1*, and *FH* pathogenic variants to increased skin cancer risk is limited. Given the possible association with MM and, to a lesser extent, NMSCs, at least one dermatologic evaluation should be considered, ideally incorporating individual clinical risk factors. In HLRCC, biannual dermatologic visits have been proposed because of the rare but possible transformation of cutaneous leiomyomas into cLMS, although there is no clear evidence that such surveillance improves outcomes [44]. A reasonable approach may be for dermatologists to assess skin cancer risk on an individual basis, provide guidance for monitoring cutaneous leiomyomas, and emphasize the need for evaluation if lesions change rather than routinely scheduling visits.

While skin phototype and UVR exposure are well-established risk factors for skin cancer [20–22], most epidemiological studies have not examined their role in hereditary mixed cancer syndromes [42–44, 67–69, 77–82, 86, 87, 94, 95]. Some exceptions exist: no link between known risk factors and skin cancers was found in LS [66]. Similarly, whether the skin site was chronically exposed to UV radiation or not did not affect skin cancer detection in *BRCA2* [80], *CHEK2* [80], or *CHEK2* 1100delC [105] pathogenic variant carriers. In contrast, common NMSCs in LS were more frequently detected in sun-exposed areas [69]. However, most studies do not specify the skin phototype or whether skin cancers arise in sun-exposed or protected areas, limiting insight into environmental and genetic interactions.

Given the uncertain impact of dermatologic surveillance on outcomes in hereditary mixed cancer syndromes, we propose it only for LFS and LS. While earlier-stage detection of skin cancer is associated with improved prognosis, evidence on the effectiveness of routine skin examinations in reducing skin cancer mortality remains mixed [124, 125]. At the general population level, some studies suggest that screening may reduce MM morbidity and mortality, whereas others report no clear benefit for MM or NMSC [124]. Targeted surveillance in high-risk populations may still be warranted [125] as regular dermatologic visits are known to improve their adherence to sun protection, facilitate earlier detection, and reduce the development of skin cancers [126–128]. Similar benefits may also apply to individuals with a genetic predisposition, which warrants further investigation.

Today, skin cancers in individuals with hereditary cancer syndromes are generally managed similarly to sporadic cases, using regular dermatologic evaluations and personalized prevention strategies. In the future, the diagnosis of a mixed hereditary cancer syndrome could influence treatment decisions. New preventive strategies for LFS-associated cancers have been proposed [129], and PARP inhibitors as well as checkpoint inhibitors show promise for treating advanced skin cancers [130–132]. Based on recent research, these therapies may be particularly relevant for hereditary cancer syndromes involving HR-DDR or MMR mutations (Fig. 1) [133–142]. These considerations highlight the need for further research on skin cancers in hereditary cancer syndromes.

The expert opinions summarized in Table 2 should be interpreted with caution, given the current low level of evidence supporting skin cancer screening in these populations. However, potential benefits highlight the need for further research on established risk factors and targeted surveillance in hereditary mixed cancer syndromes to improve early detection and patient outcomes.

**Table 2:** Our recommendations for skin surveillance in hereditary cancer syndromes

Hereditary Cancer Syndrome	Gene/Mutation	Associated skin cancers/Level of evidence	Surveillance Recommendation
Li-Fraumeni syndrome	*TP53* heterozygous	MM, NMSC, AK, MB/Strong BCC, cLMS/Moderate	Children, adolescents, and their parents: Dermatologist’s evaluation, risk assessment, sun safety education Starting at age 18: Dermatologist’s surveillance annually, treatment of premalignant lesions
Lynch syndrome	*MLH1*, *MSH2*, *MSH6*, *PMS2* EPCAM* heterozygous	SGC, KA/Strong SCC, MM/Moderate AK, MB/Low	Children, adolescents, and their parents: Dermatologist’s evaluation, risk assessment, sun safety education *MLH1, MSH2, EPCAM* carriers starting at age 18: Dermatologist’s evaluation, consider dermatologist’s surveillance** *MSH6*, *PMS2* carriers: Dermatologist’s evaluation, risk assessment
Hereditary Breast and Ovarian Cancer syndrome	*BRCA2* heterozygous	MM/Strong NMSC/Low	Dermatologist’s evaluation, risk assessment: MM and NMSC
	*BRCA1* heterozygous	MM/Moderate NMSC/Low	Dermatologist’s evaluation, risk assessment: MM and NMSC
ATM-associated hereditary cancer syndrome	*ATM* heterozygous	MM/Moderate	Dermatologist’s evaluation, risk assessment: MM
CHEK2-associated hereditary cancer syndrome	*CHEK2* heterozygous	MM/Moderate BCC/Low	Dermatologist’s evaluation, risk assessment: MM and BCC
BRIP1-associated cancer predisposition	*BRIP1* heterozygous	MM/Low	Dermatologist’s evaluation, risk assessment: MM
Hereditary Leiomyomatosis and Renal Cell Carcinoma (HLRCC)	*FH* heterozygous	cLMS/Moderate MM/Low	Dermatologist’s evaluation: primarily due to leiomyomas and cLMS but also to evaluate MM and BCC risk.

MM: malignant melanoma, SCC: squamous cell carcinoma, BCC: basal cell carcinoma, KA: keratoacanthoma, AK: actinic keratoses, MB: morbus Bowen, cLMS: cutaneous leiomyosarcoma, SGC: sebaceous gland carcinoma, NMSC: non-melanoma skin cancers in total

^*^EPCAM deletions silence MSH2, leading to a Lynch syndrome phenotype

^**^If such surveillance is considered feasible and resources are not limited, we recommend 1–2 yearly intervals

## Data Availability

No datasets were generated or analysed during the current study.
